# 107. A Phase 3, Randomized, Double-Blind Study to Evaluate the Efficacy and Safety of Oteseconazole (VT-1161) Oral Capsules versus Fluconazole and Placebo in the Treatment of Acute Vulvovaginal Candidiasis Episodes in Subjects with Recurrent Vulvovaginal Candidiasis (ultraViolet)

**DOI:** 10.1093/ofid/ofab466.107

**Published:** 2021-12-04

**Authors:** Mark G Martens, Bassem Maximos, Thorsten Degenhardt, Karen Person, Stacey Curelop, Mahmoud Ghannoum, Stephen Brand

**Affiliations:** 1 Tower Health, West Reading, PA; 2 Maximos OB/GYN, League City, Texas; 3 Mycovia Pharmaceuticals, Inc, Durham, North Carolina; 4 Case Western Reserve, Cleveland, Ohio

## Abstract

**Background:**

Recurrent vulvovaginal candidiasis (RVVC) affects nearly 138 million women globally each year. Currently there are no FDA approved treatments. The study was conducted to evaluate the efficacy of oral oteseconazole (VT-1161) in the prevention of culture-verified acute VVC episodes through Week 50 and compare the efficacy of oteseconazole and fluconazole in treatment of an acute VVC episode in RVVC subjects.

**Methods:**

219 subjects with history of RVVC (≥ 3 acute episodes within prior 12 months) were enrolled at 51 US sites. The study consisted of two phases.

Induction Phase: Subjects who presented with a vulvovaginal signs and symptoms score of ≥ 3 and positive KOH test identifying *Candida* were randomized to either:

• 600 mg oteseconazole on Day 1, 450 mg oteseconazole on Day 2 and matching placebo capsules; OR

• 3 sequential 150 mg doses (every 72 hours) of over-encapsulated fluconazole together with matching placebo capsules

Maintenance Phase: 185 subjects with resolved acute VVC infections (clinical signs and symptoms score of < 3) on Day 14 received:

• 150 mg oteseconazole or placebo weekly for 11 weeks

• then 37-week Follow-up period

**Results:**

Study achieved primary and secondary efficacy endpoints. Oteseconazole was superior to fluconazole/placebo in the proportion of subjects with ≥ 1 culture-verified acute VVC episode through Week 50 in the intent-to-treat (P < 0.001).

The average percentage of subjects with ≥ 1 culture-verified acute VVC episode through Week 50 was lower in the oteseconazole group (5.1%) compared to the fluconazole/placebo group (42.2%). Oteseconazole was noninferior to fluconazole in the proportion of subjects with resolved acute VVC infections at Day 14; 93.2% oteseconazole group, 95.8% fluconazole/placebo group.

The percentage of subjects who had ≥ 1 treatment-emergent adverse event (TEAE) was similar; oteseconazole (54%), fluconazole/placebo (64%). Most TEAEs experienced were mild or moderate severity in both groups and no drug-related SAEs or adverse effects on liver function or QT intervals.

**Conclusion:**

Oteseconazole was shown to be safe and effective in treatment of acute VVC, treatment of RVVC and prevention of recurrence of acute VVC episodes in RVVC subjects. Oteseconazole was non-inferior to fluconazole for treatment of acute VVC in subjects with RVVC.

**Disclosures:**

**Bassem Maximos, MD**, **Evofem Biosciences** (Scientific Research Study Investigator, Speaker's Bureau)**Mycovia Pharmaceutical** (Scientific Research Study Investigator)**Sage Therapeutics** (Scientific Research Study Investigator, Speaker's Bureau) **Thorsten Degenhardt, Ph.D**, **Mycovia Pharmaceuticals** (Employee, Shareholder) **Karen Person, M.S.**, **Mycovia Pharmaceuticals, Inc.** (Employee)**Mycovia Pharmaceuticals, Inc.** (Employee) **Mahmoud Ghannoum, PhD**, **Mycovia Pharmaceuticals** (Grant/Research Support, Research Grant or Support) **Stephen Brand, Ph.D**, **Mycovia Pharmaceuticals** (Employee)

